# Echocardiography for short-term mechanical circulatory support: a trans-Atlantic practical guide

**DOI:** 10.1093/ehjimp/qyaf067

**Published:** 2025-06-10

**Authors:** Hatem Soliman-Aboumarie, Christophe Vandenbriele, Luca Baldetti, Tim Balthazar, Sascha Ott, Jaime Hernandez-Montfort, Alina Nicoara

**Affiliations:** Department of Cardiothoracic Anaesthesia, Intensive Care, Mechanical Circulatory Support and Transplantation, Harefield Hospital, Royal Brompton and Harefield Hospitals, Hill End Road, Uxbridge, London UB96JH, UK; School of Cardiovascular, Metabolic Medicine and Sciences, King's College London, Denmark Hill, London WC2R 2LS, UK; Department of Cardiothoracic Anaesthesia, Intensive Care, Mechanical Circulatory Support and Transplantation, Harefield Hospital, Royal Brompton and Harefield Hospitals, Hill End Road, Uxbridge, London UB96JH, UK; Department of Cardiology, Heart Center Aalst, Aalst, Belgium; Department of Cancer and Surgery, Imperial College London, London, UK; Cardiac Intensive Care Unit, San Raffaele Hospital, Via Olgettina, 60 Milan 20132, Italy; Department of Intensive Care Medicine, University Hospital Brussels, Brussels, Belgium; Department of Cardiology, University Hospital Brussels, Brussels, Belgium; Department of Cardiac Anaesthesiology and Intensive Care Medicine, Deutsches Herzzentrum der Charité, Berlin, Germany; Department of Cardiology, Charité—Universitätsmedizin Berlin, Corporate Member of Freie Universität Berlin and Humboldt-Universität zu Berlin, Berlin, Germany; DZHK (German Centre for Cardiovascular Research ), Partner Site Berlin, Berlin, Germany; Outcomes Research Consortium, Houston, TX, USA; Advanced Heart Disease Program, Baylor Scott and White Health, Temple, TX, USA; Department of Anesthesiology, Duke University, Durham, NC, USA

**Keywords:** stMCS, cardiogenic shock, ECMO, acute cardiac care

## Abstract

Short-term mechanical circulatory support (stMCS) devices are increasingly utilized for haemodynamic stabilization in patients with cardiogenic shock. Echocardiography plays a pivotal role across the continuum of stMCS use—from patient selection and device implantation to monitoring, troubleshooting, and weaning. This review provides a comprehensive, practical guide for clinicians on the echocardiographic assessment of commonly used stMCS devices, including intra-aortic balloon pump, Impella, ProtekDuo, and veno-arterial extracorporeal membrane oxygenation. We outline device-specific contraindications, key imaging views for guiding placement, and parameters for monitoring device performance and detecting complications. The paper also introduces structured echocardiographic criteria to support decision-making during weaning and explantation. Finally, we explore emerging tools such as speckle-tracking echocardiography, 3D imaging, and artificial intelligence that may further optimize stMCS management. Through an international, multidisciplinary collaboration, this guide aims to standardize echocardiographic practice in stMCS and improve clinical outcomes in critically ill cardiac patients.

## Graphical Abstract

**Graphical Abstract:**
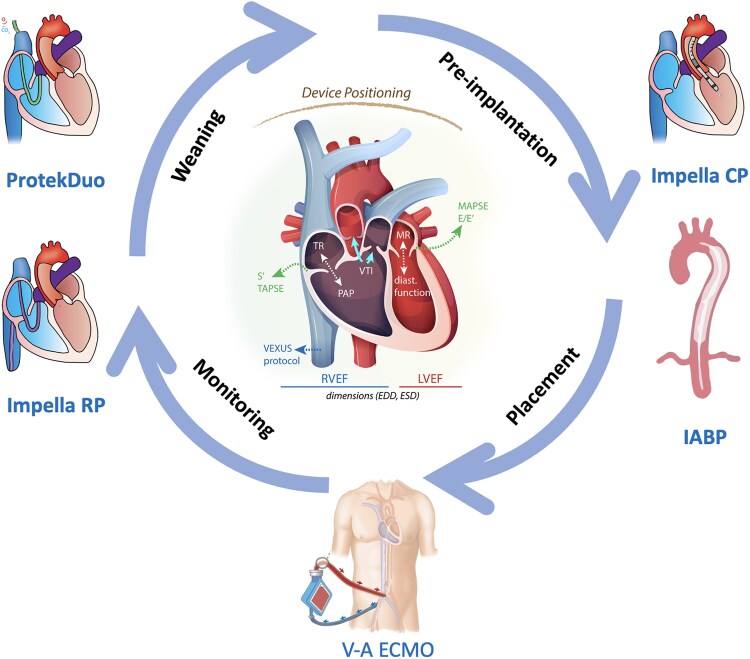
Short-term mechanical circulatory support devices and several echocardiographic parameters for assessing right and left ventricular function. Diast. Function, diastolic function; IABP, intra-aortic balloon pump; E/E′, ratio of early *trans*-mitral diastolic velocity with pulsed wave Doppler to early diastolic relaxation of mitral annulus with tissue Doppler; EDD, end-diastolic diameter; ESD, end-systolic diameter; LVEF, left ventricular ejection fraction; MAPSE, mitral annular plane systolic excursion; MR, mitral regurgitation; PAP, pulmonary artery pressure; RVEF, right ventricular ejection fraction; S′, mitral annular systolic velocity with tissue Doppler; TR, tricuspid regurgitation; TAPSE, tricuspid annular plane systolic excursion; VeXUS, Venous Excess Ultrasound score; VTI, velocity time integral; V-A ECMO, ven-oarterial extracorporeal membrane oxygenation.

## Introduction

Short-term mechanical circulatory support (stMCS) devices are increasingly utilized in acute cardiac care for the management of patients in cardiogenic shock (CS)^1^ by stabilizing haemodynamics and allowing for either myocardial recovery or long-term intervention decisions such as bridging to long-term (durable) ventricular assist devices or heart transplantation. Echocardiography is essential for the effective implementation and management of stMCS as it provides guidance during implantation, as well as information regarding device positioning, complications, and real-time longitudinal cardiac function assessment. However, despite its critical role, the use of echocardiography in stMCS management remains highly variable across institutions, with differing protocols, operator expertise, and access to advanced imaging techniques. This variability highlights an important unmet need for standardized approaches and training in echo-guided stMCS care.

This review provides a practical overview of the role of echocardiography in stMCS in the initiation, guidance, troubleshooting, and weaning, while identifying best practices and highlighting areas for future research.

## Overview of stMCS

Selecting the appropriate stMCS device requires an assessment of four key factors^2^: (i) severity of CS, (ii) univentricular vs. biventricular failure, (iii) presence of respiratory failure requiring oxygenation support, and (iv) anatomical or functional limitations or contraindications to certain devices.

**Figure 1:**
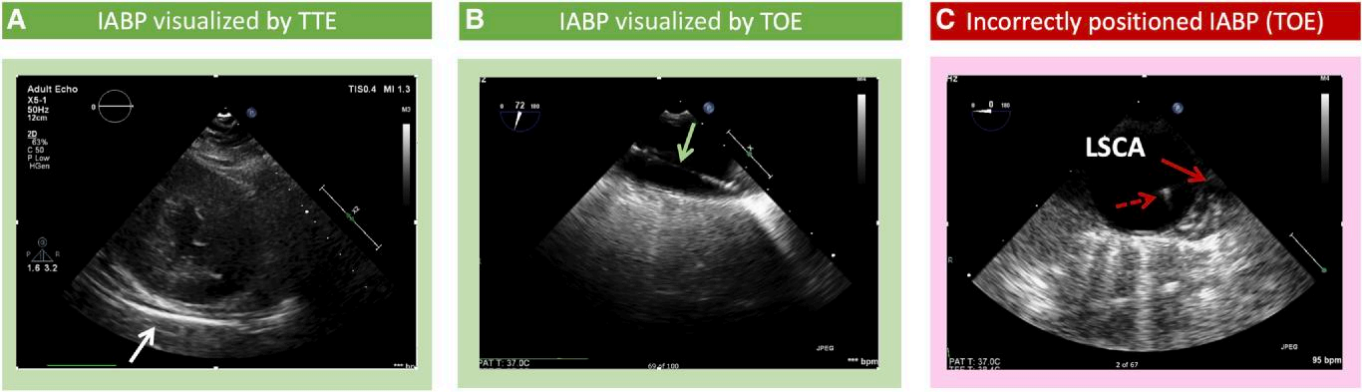
Echocardiography in IABP. (*A*) Left parasternal short-axis view showing an IABP in the descending thoracic aorta (white arrow). (*B*) TOE descending aorta long-axis view (70°) showing the IABP in its long axis (green arrow). (*C*) TOE descending aorta short-axis view (0°) showing incorrectly positioned IABP with the tip (dashed red arrow) visualized at the level of LSCA (solid red arrow).

Echocardiography is a cornerstone in the evaluation of these patients and ensuring that device selection aligns with the underlying anatomical and haemodynamic profile. In the following sections, we will briefly review the use of echocardiography in the management of some of the most used stMCS devices^3^ (*Figure 1*).

The table in the Supplementary data includes a summary of indications and specifics for each device.

## Role of echocardiography in stMCS pre-implementation

### Haemodynamic phenotyping

Echocardiography prior to stMCS is indispensable: it confirms cardiac dysfunction as the underlying driver of the circulatory shock; it allows morphologic and haemodynamic assessment of cardiac chambers—helping in the selection of the most appropriate stMCS device and identifies specific contraindications to device placement. In addition, a pre-implantation assessment provides a benchmark for comparison on repeated imaging during the clinical course of the patient, in order to track the subsequent haemodynamic trajectory. The role of non-invasive ‘echodynamic’ evaluation has shown to be reliable, except for the estimation of left atrial pressure, which remains difficult in the critically ill patients, especially in case of stMCS.^4^

Pre-implantation echocardiographic assessment should include a thorough exploration of the left and right ventricles (LV and RV) and atria, atrioventricular and semilunar valves, pericardial space, inferior vena cava (IVC), and lung fields. Notwithstanding LV ejection fraction (LVEF) as a straightforward measure of LV contractile function, we recommend to always assess LV outflow tract velocity-time integral (LVOT VTI) to non-invasively calculate LV stroke volume, as this represents a more sensitive and reliable gauge of LV ejection on repeated assessments and particularly once the stMCS has been deployed. Nevertheless, LVOT diameter should also be taken into consideration as it influences stroke volume (i.e. large LVOT diameter could compensate for reduced LVOT VTI achieving normal stroke volume). Moreover, whenever a stMCS is considered, assessment of the LV end-diastolic volume (LVEDV) and diameter (LVEDD) and of the LV diastolic function through *trans*-mitral pulsed wave Doppler and mitral annular tissue Doppler imaging is warranted. Right-sided chambers should also be explored to estimate longitudinal and radial RV function, RV outflow tract VTI, pulmonary artery (PA) pressure, and central venous pressure (CVP). Notably, assessment of right heart preload should be multiparametric, as positive end-expiratory pressure, pre-existing cor pulmonale, and severe tricuspid regurgitation (TR) can yield a dilated and non-collapsible IVC and a raised CVP despite being preload responsive. The recently proposed Venous Excess Ultrasound (VeXUS) score provides a multiparametric assessment of CVP and is more accurate than IVC dimension and collapsibility in identifying elevated CVP^5–7^ and predicts mortality in the acute heart failure setting.^8^ Lung ultrasound also plays a crucial role in assessing acute heart failure by detecting pulmonary congestion through B-lines, pleural effusions, and dynamic changes in lung aeration, providing valuable real-time information for patient management.^9^

Finally, given the strong interplay of LV and RV, the interventricular septum position and motion should always be evaluated. The degree of detail of any echocardiographic evaluation should be linked to the clinical stability of the patient, as timely and rapid stMCS may be required in highly unstable patients (e.g. Society of Cardiovascular Angiography and Interventions Class D to E patients).

### Suitability for stMCS and device-specific contraindications

Transthoracic echocardiography (TTE) and—if indicated—transoesophageal echocardiography (TOE) offer important information on patient’s suitability for the different stMCS devices.

Echocardiographers should be familiar with the absolute and relative contraindications of each stMCS device before implantation. In the absence of absolute contraindications, we should carefully look for risk indicators, which could interfere with optimum function of the stMCS devices^10^ (*Table 1*).

**Table 1:** Echocardiographic risk indicators before implantation of stMCS devices^10^

Device	Echocardiographic risk indicators
IABP	Aortic dissection and significant atheromatous disease (large, mobile plaques) >mild AR
Impella CP/5.5	Intracardiac shunt (VSD, ASD) Intracardiac thrombi LV rupture Small LV cavity Narrow LVOT diameter Significant RV dysfunction Valvular heart disease: >moderate MS, significant myxomatous MV disease, moderate AR, mechanical aortic prosthetic valve, or severe AS Aortic dissection and significant atheromatous disease Cardiac tamponade
Impella RP/ProtekDuo	Structural abnormalities of the IVC or SVC. Devices: e.g. IVC filters RA, RV, or PA thrombi or masses Valvular heart disease: mechanical prosthetic TV and PV, moderate TV or PV stenosis, or moderate PR Significant LV dysfunction Severe pulmonary hypertension
V-A ECMO	Structural abnormalities of the IVC/RA Devices: e.g. IVC filters. Atrial septal aneurysm Intracardiac shunt: ASD or VSD Valvular heart disease: >moderate AR Aortic dissection and significant atheromatous disease

AR, aortic regurgitation; AS, aortic stenosis; ASD, atrial septal defect; ECMO, extracorporeal membrane oxygenation; IABP, intra-aortic balloon pump; IVC, inferior vena cava; LV, left ventricle; LVOT, left ventricular outflow tract; MS, mitral stenosis; MV, mitral valve; PA, pulmonary artery; PR, pulmonary regurgitation; PV, pulmonary valve; RA, right atrium; RP, right percutaneous (Impella RP); RV, right ventricle; SVC, superior vena cava; TV, tricuspid valve; V-A ECMO, veno-arterial extracorporeal membrane oxygenation; VSD, ventricular septal defect.

#### IABP

**Figure 2:**
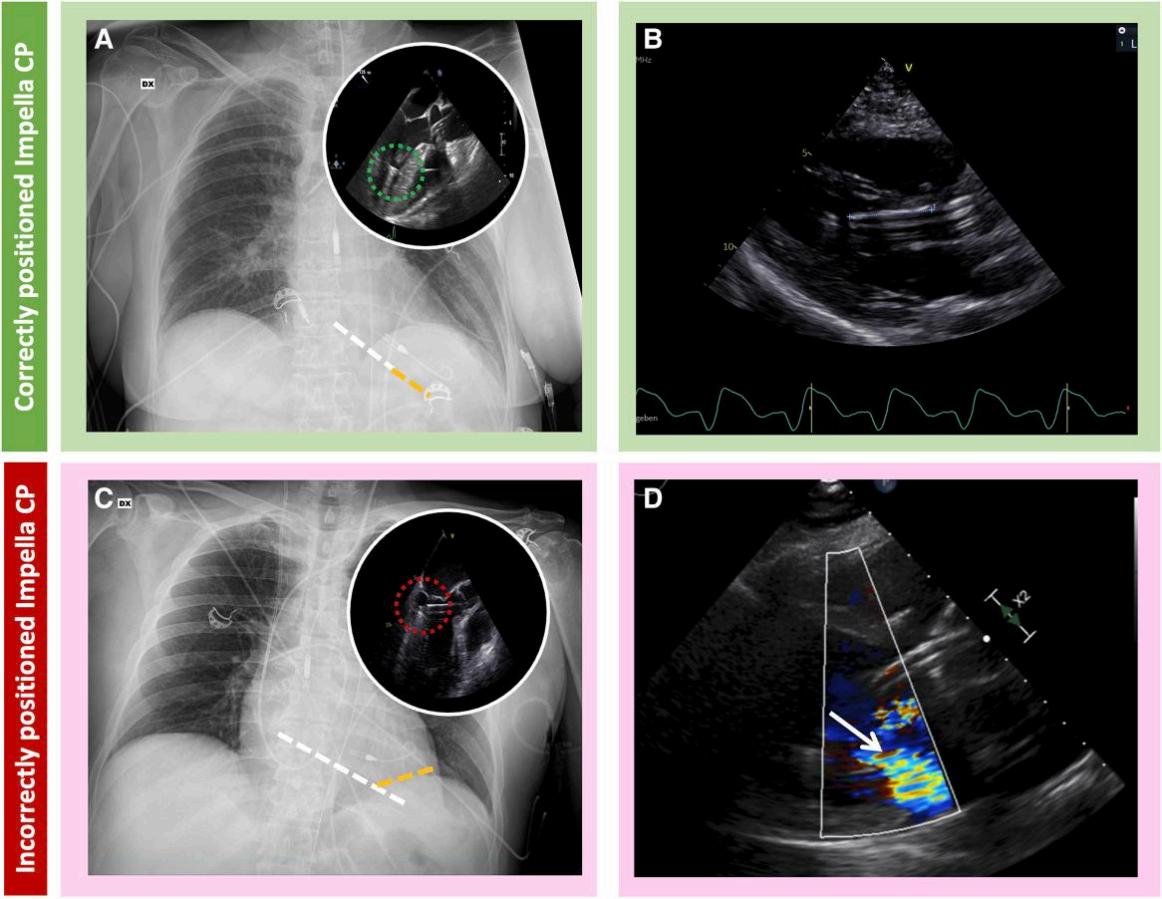
Echocardiography in Impella CP. (*A*) Correctly positioned Impella CP with chest radiography showing the major axis of the distal Impella shaft (white dashed line) and the pigtail axis (yellow dashed line); therefore, the pigtail bending angle is 0. TOE in the same panel is showing mid-oesophageal long-axis view at 120° with the Impella inlet (green dashed circle) in the apex. (*B*) Left parasternal long-axis view showing a correctly positioned Impella CP with the inlet located 4 cm from the aortic valve annulus. (*C*) Malrotated Impella CP with chest radiography showing the major axis of the distal Impella shaft (white dashed line) and the pigtail axis (yellow dashed line); therefore, the pigtail bending angle is 45°. TOE in the same panel is showing mid-oesophageal long-axis view at 120° with the Impella inlet impinging on the LV inferolateral wall (red dashed circle). This example demonstrates a crushed pigtail sign. (*D*) Left parasternal long-axis view showing an incorrectly positioned Impella CP with the device impinging on the anterior mitral leaflet leading to MR (white arrow).

Echocardiographic assessment is crucial to identify contraindications for intra-aortic balloon pump (IABP) (*Figure 2*). Moderate or greater aortic regurgitation (AR) can reduce the haemodynamic benefits of counterpulsation and exacerbate LV dilation and pulmonary congestion. Significant aortic atheroma increases the risk of balloon rupture, embolism, or atheroma dislodgment, necessitating TOE assessment of the aortic valve and thoracic aorta. IABP is ineffective in isolated RV failure and may worsen systemic hypotension due to insufficient LV preload.^11,12^ Additionally, it can aggravate vasoplegia or dynamic LVOT obstruction by reducing afterload. Anatomical contraindications, such as aortic dissection or coarctation, must also be ruled out. However, successful use in patients with Type A dissection has been reported following echocardiographic confirmation of placement in the true lumen.^13^ Therefore, while IABP is effective in select cases, careful echocardiographic evaluation is essential to optimize patient selection and avoid potential complications.

#### Left-sided microaxial flow devices (Impella CP/5.5)

**Figure 3:**
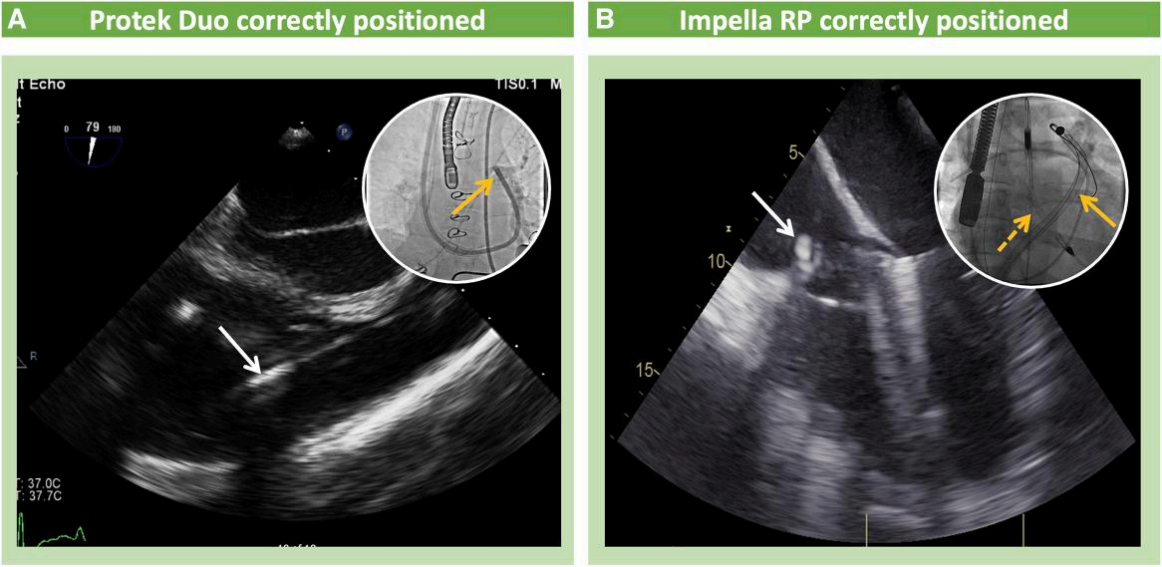
Echocardiographic guided positioning of Impella RP and ProtekDuo. (*A*) TOE assessment of the ProtekDuo cannula. (*A*) Modified mid-oesophageal RV inflow–outflow view showing the device (white arrow) extending into the RV and across the pulmonary valve into the PA. Fluoroscopy anteroposterior view of the same device (white-framed circle) seen entering the RA via the SVC with the device outflow in the PA (yellow arrow). A PA catheter (white arrow) from the right internal jugular vein is also seen. (*B*) TOE assessment of the Impella RP device. Mid-oesophageal four-chamber view showing the device’s body angle (white arrow) traversing the TV valve. (*B*) Fluoroscopy anteroposterior view of the same device shows Impella RP device (yellow arrow) positioned in the RV from the IVC and the Impella CP device (yellow dashed arrow) (BiPELLA configuration).

The left-sided Impella (*Figure 3*) offers effective LV unloading and improved systemic perfusion; therefore, they are suitable for patients with isolated LV failure. Isolated or predominant RV failure should be ruled-out, as the left-sided Impella forward flow might aggravate the RV failure due to RV/Impella flow mismatch. In addition, in case of LV underfilling due to insufficient RV output, the left-sided Impella could precipitate LV chamber collapse, resulting in suction events and haemolysis. Traditional echocardiographic predictors of RV failure after initiation of left-sided durable continuous-flow support include a TAPSE < 7.5 mm and increased RV dimensions relative to the failing LV (i.e. RV to LV diameter ratio > 0.75). This rationale extends also to the risk stratification of RV failure after initiation of Impella support.^14,15^ In addition, a paradoxical leftward interventricular septum shifting suggests a prevailing RV dysfunction contributing to the clinical picture. To ensure adequate support, aortic valve competence is required, as significant AR may lessen antegrade blood flow and increase LV end-diastolic pressure due to blood recirculation between aorta and LV. Therefore, whenever more than moderate AR is observed, initiation of left-sided Impella should be considered on a case-by-case basis and weighted against other stMCS. Notably, Impella insertion has been safely and effectively performed in cases of both severe aortic stenosis, aortic bioprosthesis, and AR.^16,17^ Additional selected echocardiographic markers of higher risk for device-related complications include a small LV cavity and a mitro-aortic angle < 126.5°—all associated with a higher risk of suction and haemolysis.^18^ Conversely, a larger LV (LVEDD > 50.5 mm) conveys an increased risk of Impella device malrotation within the LV.^19^ Anatomical contraindications to Impella insertion include the presence of mechanical aortic prosthesis, aortic dissection, and LV mobile thrombi, all of which can be identified on echocardiography.

#### Right-sided stMCS devices (ProtekDuo/Impella RP)

Right-sided stMCS devices have somehow less availability compared with left-sided stMCS devices. When evaluating the use of RV support devices in patients with (isolated) RV failure, TTE and TOE play a crucial role in identifying potential contraindications or factors that may lead to device dysfunction. These include the presence of thrombi in the right atrium (RA) or RV, tricuspid or pulmonary valve stenosis, mechanical tricuspid or pulmonary prosthetic valves, or intracardiac shunts (such as atrial septal defect, ventricular septal defect, or patent foramen ovale) that could result in left-to-right shunting. Furthermore, echocardiography should evaluate pulmonary valve competence, as significant pulmonary regurgitation (PR) would decrease the flow to the pulmonary circulation and the RV unloading effect of the device. In addition, the presence of prominent, floating atrial structures (e.g. Chiari network and/or Eustachian valve) could interact with the cannula placement and interfere with the atrial drainage. Finally, echodynamic assessment should rule out severe pulmonary hypertension and signs of increased left atrial pressure as the first would diminish the forward flow generated by the ProtekDuo cannula at any given speed and the latter could precipitate pulmonary oedema as the antegrade flow of the device will worsen left chamber and pulmonary circulatory stasis. Tricuspid valve function should be assessed using 2D, color flow doppler, and spectral Doppler echocardiography.^20^ While TR is typically well tolerated during temporary right ventricular assist device support, the presence of more than mild PR may limit the efficacy of device outflow delivered to the PA.^20^ Of note, the presence of masses in the right heart chambers or PA could lead to device obstruction or pulmonary embolism.^20^

#### V-A ECMO

Echocardiography plays a crucial role in identifying or ruling out reversible cardiac pathologies that may be the primary cause of haemodynamic deterioration, such as cardiac tamponade, undiagnosed valvular disease, or LV dysfunction. It is also essential for detecting absolute contraindications to veno-arterial extracorporeal membrane oxygenation (V-A ECMO), such as aortic dissection. Significant AR is a relative contraindication to V-A ECMO, as the increased LV afterload can exacerbate AR, worsening LV distension and pulmonary congestion. Even moderate AR can cause significant LV distension and congestion at high V-A ECMO flows.^21^ Additionally, echocardiography provides valuable insights into aortic atherosclerosis, guiding the imaging specialist in selecting the optimal cannulation approach (central vs. peripheral) and technique (surgical vs. percutaneous) to minimize complications. Furthermore, it is instrumental in assessing right heart morphology, helping to identify structural abnormalities that could impede venous cannula placement, and ensuring effective device function and patient safety.

Severe, spontaneous echo contrast is another high-risk marker for LV overload, stasis, and subsequent thrombosis.^22^ Finally, severe mitral regurgitation (MR) intrinsically carries a higher risk for pulmonary oedema and might be worsened by the retrograde V-A ECMO flow.

## Role of echocardiography during stMCS

### Guidance for device placement

#### IABP

The efficient operation of an IABP relies on proper positioning, with the balloon tip placed ∼2 cm distal to the origin of the left subclavian artery (LSA) and the proximal end positioned above the diaphragm (*Figure 2*). Echocardiographic assessment and guidance can be performed using TTE (off-axis apical two-chamber and suprasternal views)^23^ or TOE; however, TOE is often preferred for superior visualization of guidewire navigation in the aorta. Advancement of the femorally inserted guidewire can be monitored using short-axis and long-axis aortic views. The IABP tip should be positioned just below the origin of the LSA, which can be visualized by counterclockwise rotation of the TOE probe from an upper oesophageal aortic arch short-axis view, where the LSA appears at the 1 o’clock position. To position the IABP correctly, the echo-dense distal tip of the catheter and the lucent balloon body are distinguished from the thin guidewire. The origin of the LSA is identified, and the balloon tip is advanced to ∼2 cm below this point. A simple confirmation technique involves locating the balloon tip in a descending aorta short-axis view, placing the echocardiographer’s fingertips on the TOE probe at the patient’s teeth, and withdrawing the probe until the LSA origin appears. The distance from the fingers to the teeth represents the distance from the balloon tip to the artery, guiding final adjustments. If the LSA origin is not visible, the inferior margin of the aortic arch serves as an alternative landmark, with the IABP tip positioned just distal to this point. After final positioning, ensure there is no occlusion of the LSA or left carotid artery.

#### Left-sided micro-axial flow pump

Proper positioning of the guidewire and Impella in the LV should account for individual intracardiac anatomical variations (*Figure 3*). Accurate initial positioning is essential for optimal device function, preventing complications such as suction events and haemolysis, and facilitating later correction in case of inlet malrotation. Device insertion depends on the Impella type: Impella CP is typically implanted percutaneously via the femoral artery, while Impella 5.5 is inserted surgically via the axillary artery or direct aortic approach. Guidewire visualization in the aorta and exclusion of aortic dissection are best accomplished using TTE parasternal short- and long-axis aortic views for femoral access. Wire crossing of the aortic valve and intraventricular positioning of the wire, followed by the Impella, is best achieved using the mid-oesophageal long-axis view (or parasternal long-axis view in TTE). Misplacement of the wire across the mitral valve must be identified and corrected. The device inlet should be positioned freely within the LV cavity, directed towards the apex, without obstruction from the papillary muscle or mitral valve. After device placement, the guidewire is removed, and the final insertion depth is set. Recommended depths (measured from the device inlet to the aortic valve) are ∼3.5 cm for Impella CP and 4.5–5 cm for Impella 5.5, with slight adjustments based on individual anatomy to ensure optimal pump performance. The outlet area should be seen well above the aortic valve. The positions of the inlet and outlet zones can be inferred based on the artefacts they produce. In the mid-oesophageal long-axis view, the inlet zone, located in the LV far field, appears as a hypoechoic area due to acoustic shadowing. Meanwhile, the outlet area, seen in the near field within the ascending aorta, generates a prominent reverberation artefact.^20,24^

Additionally, when the device is correctly positioned, colour flow Doppler interrogation should reveal a mosaic pattern of turbulence around the outlet area, well above the aortic valve, confirming appropriate flow dynamics.^20,24^

In addition to device depth, proper orientation within the LV should always be ascertained. The device inlet should be positioned freely within the LV cavity, directed towards the apex. The abnormal orientation of Impella inlet and pig-tail away from the LV apex and towards the mitral valve inlet and the inferolateral LV wall has been termed ‘Impella malrotation’ and is associated with poor outcomes and worse LV unloading in CS patients.^19,25^ Echocardiography promptly detects malrotation and should systematically be done at insertion and at least daily during support. In addition, the presence of the ‘crushed pig-tail sign’ (i.e. an abrupt bend of the Impella pigtail) on chest X-ray can also suggest malrotation^26^ (*Figure 3*).

#### Right-sided stMCS devices

##### ProtekDuo/Impella RP flex

**Figure 4:**
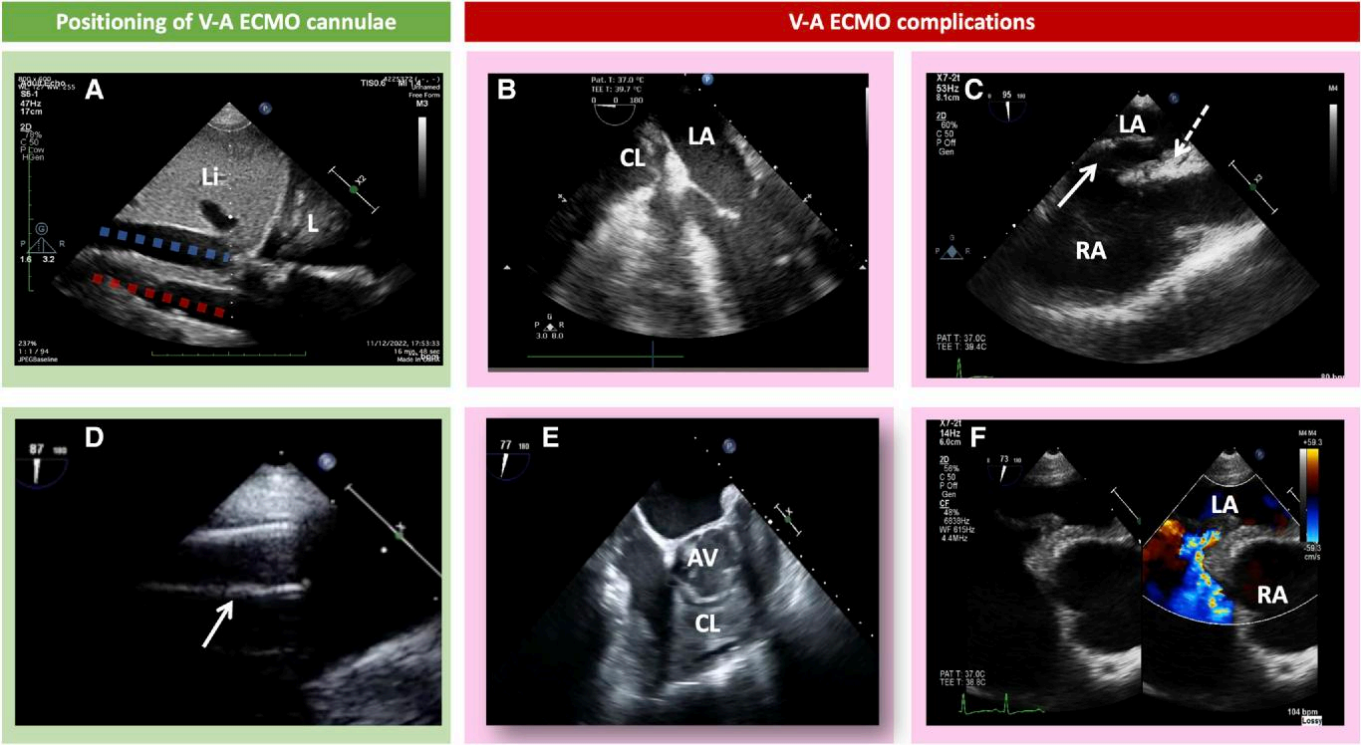
Echocardiography for positioning and troubleshooting during V-A ECMO complications. (*A*) Trans-hepatic mid-axillary window showing the IVC (blue dashed line) and the abdominal aorta (red dashed line) underneath the liver (Li) and the right lung (L). This view is useful for confirming guidewire positioning for both femoral venous and arterial cannula during peripheral V-A ECMO cannulation. (*B*) Mid-oesophageal four-chamber view at 0° showing a collection (CL) compressing the RA, which impaired V-A ECMO flow. (*C*) Mid-oesophageal bicaval view at 95° showing ECMO venous drainage cannula (white arrow) inadvertently invaded the interatrial septum (white dashed arrow). (*D*) Mid-oesophageal bicaval view at 87° showing the ECMO venous cannula (white arrow) tip correctly positioned in the subhepatic IVC. (*E*) Mid-oesophageal RV inflow–outflow view at 77° showing a clot (CL) in the RA extending into the PA in a patient on peripheral V-A ECMO. (*F*) Colour Doppler of the same window and same patient in *C* showing interatrial shunt induced by inadvertent venous cannula malpositioning, which infiltrated the interatrial septum and led to an iatrogenic interatrial septal defect. The patient required IAS defect closure with a septal closure device before V-A ECMO weaning.

TOE with colour Doppler is highly effective in evaluating the orientation of the outflow jet and the positioning of the outflow cannula (*Figure 4*). The cannula tip should be positioned 1–2 cm beyond the pulmonary valve within the main PA, ensuring that the flow is directed forward. The side holes at the distal end of the cannula facilitate optimal flow distribution into both the right and left PAs, promoting efficient circulation.^27^

For verification, several mid-oesophageal TOE views can be used, including the mid-oesophageal RV inflow outflow and upper oesophageal aortic arch short-axis views with colour Doppler and continuous-wave Doppler.

##### Impella RP

The device is positioned across the tricuspid and pulmonary valves, with the inflow port located in the IVC and the outflow port in the PA. Ideally, the outflow port should be positioned in the main PA, directed towards the left PA, allowing the Swan–Ganz catheter to be placed in the right PA.^24^ A bicaval view on TOE can help visualize the inflow port, typically positioned at the IVC/RA junction, while a mid-oesophageal RV inflow–outflow view and upper oesophageal views can confirm proper outflow port placement. Similar to left-sided Impella devices, the Impella right percutaneous (RP) is susceptible to migration, which may result in device malfunction. If the outflow port is positioned at or below the pulmonary valve, it can lead to reduced support or even complete recirculation within the RV. In such cases, TTE or TOE should be used to verify proper Impella positioning.^28^

#### V-A ECMO

After cannulating the femoral vein and/or artery, the guidewire’s passage into the IVC and/or aorta can be verified using TTE (mid-axillary transhepatic view) or TOE. The IVC is best visualized by advancing the probe from a modified mid-oesophageal bicaval view while the thoracic aorta can be assessed using the descending aorta long- and short-axis view.^29^

**Figure 5:**
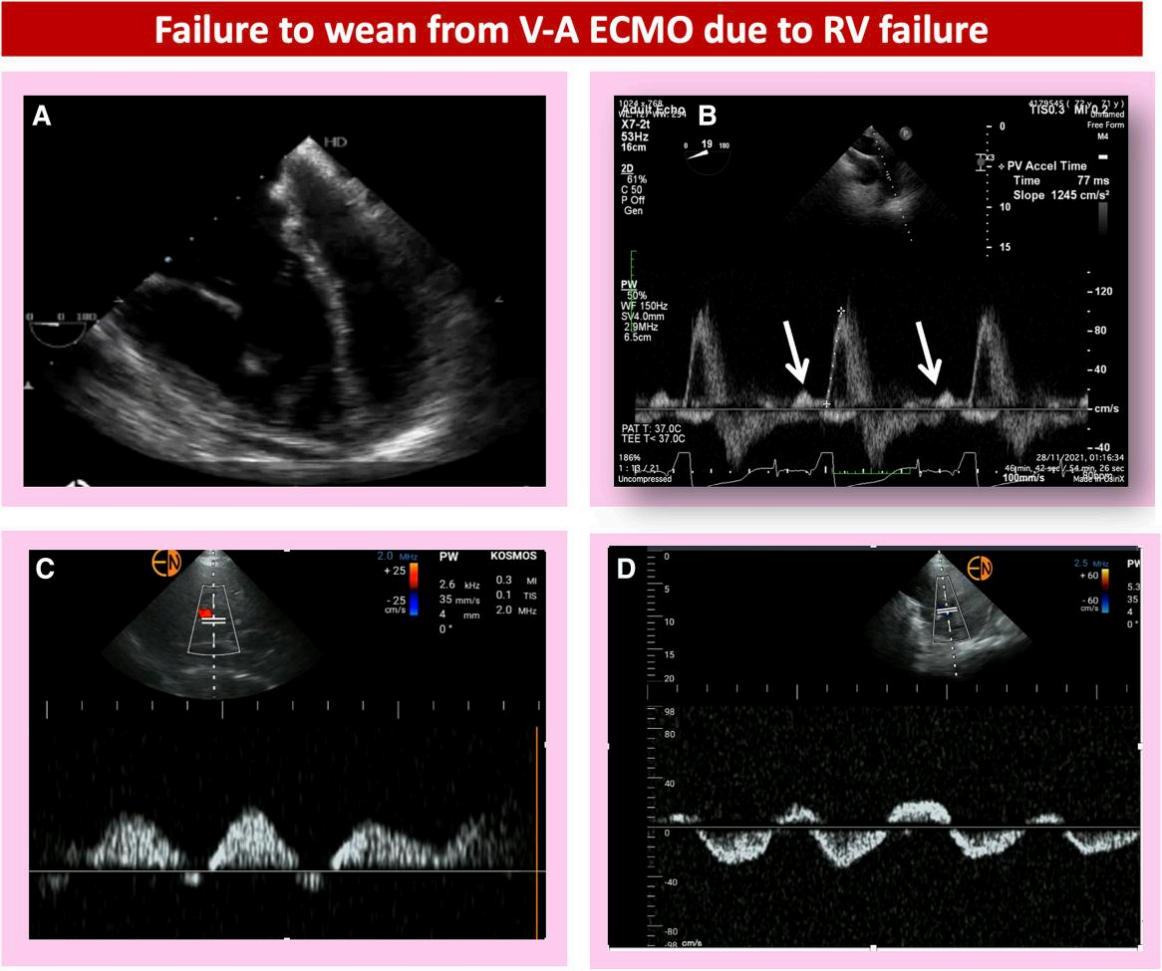
Echocardiographic and point of care ultrasound assessment of the right heart in a patient who failed weaning from V-A ECMO. (*A*) Mid-oesophageal four-chamber view at 0° showing significantly dilated RV with shift of the interatrial septum and interventricular septum to the left side. (*B*) Upper oesophageal ascending aorta view with pulsed wave Doppler interrogation of the PA showing shortened acceleration time (77 ms) and a pre-systolic A wave (white arrows), which indicates restrictive RV physiology in the context of elevated pulmonary vascular resistance. The pre-systolic A wave occurs when RV end-diastolic pressure exceeds PA diastolic pressure; hence, atrial contraction will lead to pulmonary valve opening. (*C*) Pulsed wave Doppler assessment of the portal vein in the same patient is showing significant portal vein pulsatility (>100%), which is a severely abnormal finding and indicates a significantly elevated RV preload (systemic venous congestion). (*D*) Pulsed wave Doppler assessment of the hepatic vein in the same patient is showing systolic flow reversal, which is a severely abnormal finding and indicates a significantly elevated RV preload (systemic venous congestion).

When inserting the guidewire via the right internal jugular vein, its passage through the superior vena cava (SVC) and into the RA can be confirmed in the mid-oesophageal bicaval view (*Figure 5*). Once proper guidewire placement is confirmed, percutaneous dilation and ECMO cannula insertion can be performed. The drainage cannula can then be advanced up the IVC into the RA, and femoral arterial cannula is not usually seen on TOE.

### Monitoring bedside device performance and troubleshooting in the intensive care unit

Multidisciplinary echo rounds might be valuable in daily bedside monitoring, assessing device performance and troubleshooting the following:

#### Assessing LV unloading and decompression

V-A ECMO configuration poses the risk of increased LV afterload, potentially reducing LV ejection to the point of persistent, complete closure of the aortic valve.^30^ Serial assessment of LVOT VTI and visual assessment of aortic valve opening while increasing V-A ECMO flow help to identify worsening LV ejection and prompt venting strategies.^31^

Echocardiographic parameters of LV unloading in stMCS have been predominantly studied in durable continuous-flow LV assist devices.^32^ Key parameters with reasonable accuracy in distinguishing PCWP above or below 15 mmHg include right atrial pressure, systolic pulmonary artery pressure, left atrial volume index, E/A, and E/e′. Additionally, a relative change in LVEDd has been identified as a useful metric.^31^ However, individually, these parameters exhibit only moderate sensitivity and specificity. The presence of severe MR, lack of aortic valve opening, and spontaneous echo contrast in the LV are indicators of inadequate LV unloading. Data specific to stMCS remain limited, although ongoing studies on speckle tracking show promise pending further validation.^33^

#### Detecting complications

Different stMCS modalities exhibit varying complication rates.^34^ Daily focused echocardiographic assessments are advised to ensure correct device positioning and detect complications, including intracardiac or valvular thrombi and cardiac tamponade (*Table 2*). Haemodynamics of tamponade are modified because of ECMO and the effects of it may only become apparent when reducing the flow during a weaning trial.^35^ A pericardial haematoma that compresses cardiac chambers may not change haemodynamics if it does not affect cannula flow

**Table 2:** Echocardiographic assessment during stMCS complications

Complication	Recommended echocardiographic view	Assessment
Intraaortic balloon pump		
Distal embolization/stroke	TOE: ME descending aorta LAX/SAX	Check thrombus formation around balloon
Insufficient LV unloading	TTE: PS LAX, AP4C TOE: ME 4C, ME MC, TG 2C	LVEDd, LAVi, E/A, E/e′ Mitral regurgitation
Microaxial flow pump (Impella)		
Suctioning: Diastolic suction: hypovolaemia/filling status Continuous suctioning: device malposition/malrotation, RV dysfunction, cardiac tamponade	TTE: PS LAX, AP4C, SX 4C TOE: ME 4C, ME LAX, ME RV OT, modified ME bicaval view.	Assess volume status Check RV and IVC Assess device position and RV function Check for pericardial effusion
Haemolysis	TTE: PS LAX, AP4C, PS SAX. TOE: ME 4C, ME LAX, ME RVOT, modified ME bicaval view	Device position RV function Intracardiac Thrombi
Device malposition	TTE: PS LAX TOE: ME LAX	Assess insertion depth of device
Insufficient LV unloading	TTE: PS LAX, AP4C TOE: ME 4C, ME MC, TG 2C	LVEDd, LAVi, E/A, E/e′ Mitral regurgitation
V-A ECMO		
Insufficient LV unloading	TTE: PS LAX, AP4C TOE: ME 4C, ME MC, TG 2C	LVEDd, LAVi, E/A, E/e′ Mitral regurgitation
Suctioning Hypovolaemia Cannula malposition	TTE: PS LAX, AP4C, SX 4C TOE: ME 4C, ME LAX, ME RV OT, modified ME bicaval view.	Volume status: RV, IVC^a^ Cannula position in RA/IVC

AP4C, apical four-chamber view; E/A, ratio of early (E) to late (A) mitral inflow velocities; E/e′, ratio of early mitral inflow velocity to early mitral annular velocity; IVC, inferior vena cava; LAX, long-axis view; LAVi, left atrial volume index; LV, left ventricle; LVEDd, left ventricular end-diastolic diameter; ME MC, mid-oesophageal mitral commissural view; ME LAX, mid-oesophageal long-axis view; ME RV OT, mid-oesophageal RV outflow view; PS SAX, parasternal short-axis view; RA, right atrium; RV, right ventricle; RVOT, right ventricular outflow tract; SX, subcostal view, TG 2C, transgastric two-chamber view; V-A ECMO, veno-arterial extracorporeal membrane oxygenation.

^a^Evaluation of IVC might be influenced by ECMO drainage cannula.

## Role of echocardiography during stMCS weaning

### Weaning

**Figure 6:**
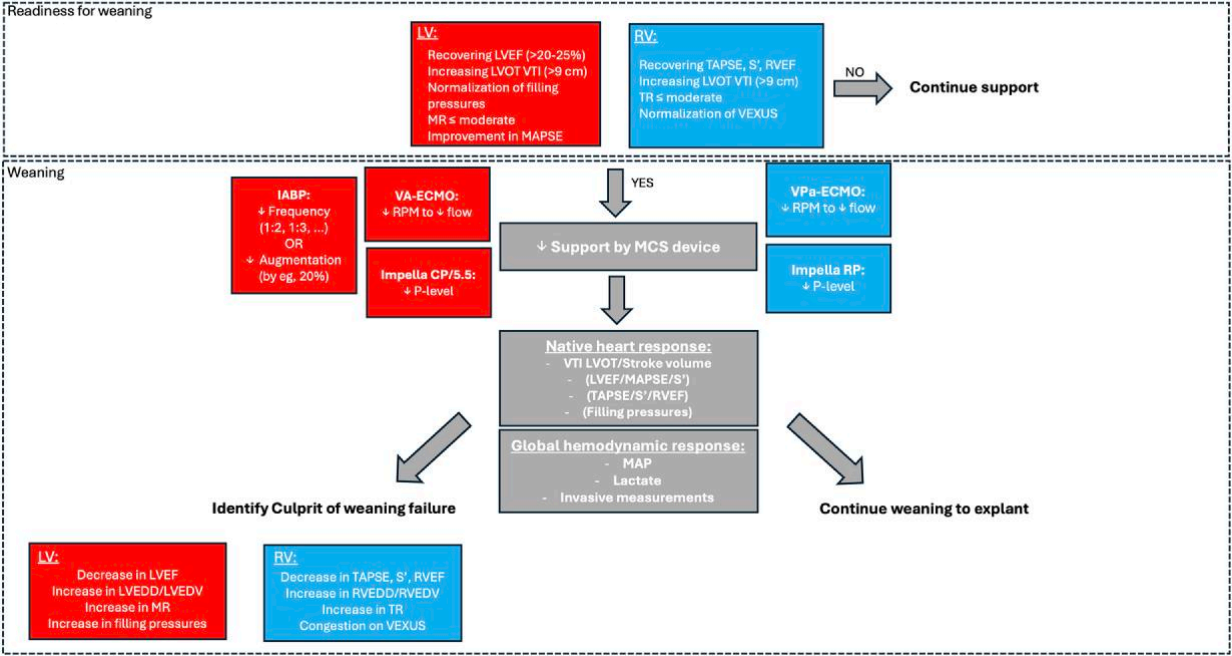
Schematic illustration of readiness for weaning and a proposed weaning algorithm per stMCS-device.

**Figure 7:**
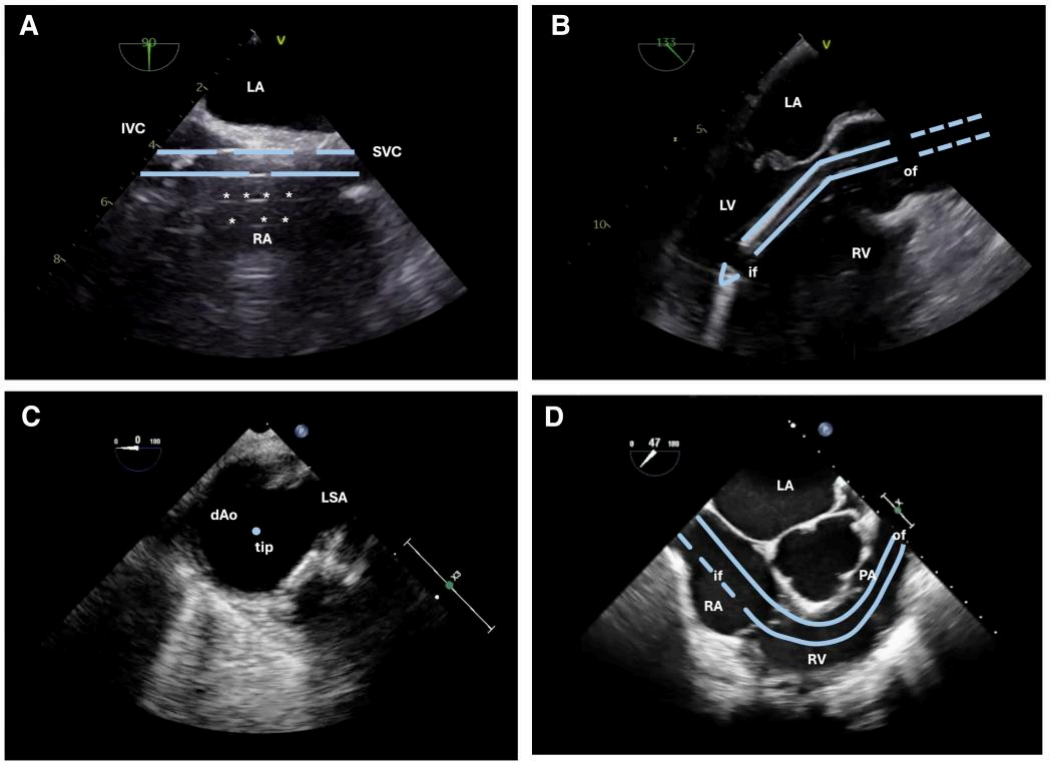
Schematic illustration of tMCS cannula positions. (*A*) venous drainage cannula of V-A ECLS System in TEE ME bicaval. *Note*: typical reverberation artefacts (*). (*B*) Impella 5.5 in TEE ME long axis. (*C*) IABP tip in TEE Ao SAX. *Note*: the tip of the IABP should be just below the origin of the LSA. (*D*) ProtekDuo cannula in TEE RV inflow–outflow. IVC, inferior vena cava; SVC, superior vena cava; LA, left atrium; RA, right atrium; LV, left ventricle; RV, right ventricle; if, inflow; of, outflow; dAo, descending Aorta; LSA, left subclavian artery; PA, pulmonary artery.

stMCS weaning is the gradual reduction of the level of support to a minimum level with the goal of either de-escalation or explantation. However, before initiating the weaning process, it is essential to evaluate the patient’s readiness. ‘Readiness to wean’ indicates that the patient has demonstrated sufficient recovery across clinical, haemodynamic, metabolic, and imaging parameters to warrant a trial of reduced circulatory support, referred to as a weaning trial. Weaning from stMCS is primarily a clinical decision and does not always require full restoration of cardiac function. The most studied echocardiographic parameters for assessing readiness to wean are LVEF ≥ 20–25% and LVOT VTI ≥ 9–10 cm. However, these parameters are highly influenced by global haemodynamics, and observing an improving trend is often more critical than meeting absolute thresholds.^35,36^ Additionally, structural considerations play a key role in readiness to wean. Persistent severe MR or other structural abnormalities requiring intervention should be thoroughly evaluated before initiating the weaning process. A comprehensive echocardiographic assessment helps identify residual issues that could impact the success of support de-escalation or explantation (*Figures 6* and *7*).

The ultimate goal is to predict stability after decannulation (ready to decannulate). This process varies depending on the specific device being used. *Table 3* summarizes echocardiographic parameters used during weaning of MCS.

**Table 3:** Echocardiographic parameters for assessing weaning/de-escalation from stMCS

	Readiness to wean (on normal flow)	Readiness to explant (at low flow)	
IABP	Recovering LVEF (≥20–25%) Increasing LVOT VTI (≥9 cm) Normalization of left-sided filling pressures MR ≤ moderate	LVEF ≥ 20–25% LVOT VTI ≥ 10 cm Stable diastolic function MR ≤ moderate	
Impella	Recovering LVEF (≥20–25%) Increasing LVOT VTI (≥9 cm) Normalization of filling pressures MR ≤ moderate	LVEF ≥ 20–25% LVOT VTI ≥ 10 cm S′ ≥ 6 cm/s Stable diastolic function MR ≤ moderate	
V-A ECMO	Recovering LVEF (>20–25%) Increasing LVOT VTI (>9 cm) Normalization of filling pressures MR ≤ moderate Improvement in MAPSE Improvement in TAPSE	LV: LVOT VTI ≥ 10 cm (37) LVEF ≥ 20–25% (37) S′ ≥ 6 cm/s (55) (39) LV strain increase Increase in lateral e′ (37) LVETc ≥ 208 ms (37) LVETc/PAWP > 15.9 (37) Neutral position of IVS	RV: S′ increase by 10% RVEF ≥ 24% (40) TAPSE/sPAP > 0.45 RVFWS/SPAP > 0.45 S′/sPAP > 0.33
ProtekDuo/Impella RP	Improvement in TAPSE, RV S′, RV FAC Neutral position IVS VeXUS Grade 1	LVOT VTI ≥ 10 cm No deterioration in TAPSE, S′, FAC, RVEF Neutral position IVS maintained VeXUS Grade 1 maintained	

e′, early diastolic mitral annular velocity; FAC, fractional area change; IABP, intra-aortic balloon pump; IVS, interventricular septum; LV, left ventricle; LVEF, left ventricular ejection fraction; LVETc, corrected left ventricular ejection time (LV ejection time measured in M-mode in parasternal long-axis view and divided by the square root of the RR interval); LVOT VTI, left ventricular outflow tract velocity-time integral; MAPSE, mitral annular plane systolic excursion; MR, mitral regurgitation; PAWP, pulmonary artery wedge pressure; ProtekDuo, dual-lumen percutaneous right ventricular assist device; RP, right percutaneous; RVEF, right ventricular ejection fraction; RV, right ventricle; RVFWS, right ventricular free wall strain; S′, peak systolic velocity of the tricuspid/lateral mitral annulus; sPAP, systolic pulmonary artery pressure; TAPSE, tricuspid annular plane systolic excursion; VeXUS, Venous Excess Ultrasound score; VTI, velocity-time integral; V-A ECMO, veno-arterial extracorporeal membrane oxygenation.

## Future directions

Speckle-tracking echocardiography (STE) and 3D echocardiography have become well-established techniques, offering significant incremental value beyond conventional 2D imaging in assessing cardiac function, monitoring myocardial recovery, and guiding stMCS weaning. STE-derived myocardial strain is particularly useful for evaluating LV unloading, as demonstrated by Hammoudi *et al*.,^33^ who reported a strong linear relationship between STE strain and LV stroke work in post-myocardial infarction pigs undergoing acute LV unloading with Impella. Similarly, in a right coronary artery embolization-induced CS model, STE provided detailed insights into global and regional LV and RV function, distinguishing the effects of vasoactive therapy from mechanical support via Impella RP.^37^

In the context of stMCS weaning, myocardial recovery can be effectively monitored using LV and RV STE and 3D parameters, which have emerged as predictors of successful de-escalation.^38,39^ These techniques are also valuable for assessing myocardial viability, informing decisions on stMCS escalation, de-escalation, or transition to long-term support. While STE has been extensively used to evaluate myocardial viability in ischaemic heart disease, its application in stMCS remains an area for further exploration.^40,41^ Given the challenges of performing cardiac magnetic resonance in critically ill patients, STE could serve as a viable alternative for assessing myocardial function in this population.

Another promising innovation is the integration of pressure-strain loops, which combine STE strain measurements with pressure data to assess myocardial work indices, providing non-invasive insights into ventricular-arterial-device coupling.^42^ Additionally, blood speckle imaging (BSI) has shown potential in stMCS management by directly tracking blood velocity vectors. BSI has been particularly useful in visualizing Impella inlets and identifying the watershed region in VA-ECMO, which represents the interface between antegrade flow from Impella and retrograde flow from ECMO. Continuous monitoring of the watershed region’s progression may offer valuable insights into readiness for ECMO weaning, making it an exciting avenue for future research.^43^

Beyond advanced functional imaging, ultrasound-enhancing agents (UEAs) have become integral in critically ill patients, particularly in post-cardiac surgery scenarios, where mediastinal changes, chest tubes, and mechanical ventilation can impair conventional echocardiography.^44^ The feasibility of UEA use in stMCS settings has been demonstrated in small studies, where it successfully identified the watershed area in the abdominal aorta in VA-ECMO patients and improved thrombus detection and LV function assessment without complications.^45^ However, safety concerns persist, particularly regarding the potential interference with arterial bubble sensors that may trigger automatic pump shutdowns. To mitigate these risks, the American Society of Echocardiography now recommends using UEAs in ECMO-supported patients only under the supervision of experienced perfusionists and clinicians.^10^

Artificial intelligence (AI) is transforming the management of stMCS by enabling real-time data analysis and supporting clinical decision-making. AI models can leverage complex patterns within patient data to identify risks and optimize treatment strategies throughout the stMCS continuum, including patient selection and risk stratification, device optimization and performance monitoring, and complication prediction and management. Machine learning (ML), encompassing supervised and unsupervised algorithms, has garnered significant attention in CS research.^46^ ML enables the analysis of large datasets, including patient demographics, clinical biomarkers, imaging data, and comorbidities, to identify CS phenotypes and predict outcomes.

A particularly promising innovation is the concept of a digital twin, a virtual model of a real-life patient that receives real-time updates on physiological and treatment data. This ‘digital twin’ can simulate and personalize clinical outcomes using AI predictions, assisting clinicians in therapy regulation and long-term prognosis adjustments.^47^ Despite its potential, several challenges need to be addressed before AI can be fully realized in the echo management of stMCS. These include the need for standardized, high-quality data to train robust AI models, ensuring the safety and efficacy of algorithms, and addressing concerns around patient privacy, algorithm bias, equity in access, and the transparency of AI systems.

## Conclusions

Echocardiography plays a pivotal role in the selection, guidance, monitoring, and escalation/de-escalation of stMCS devices, providing real-time haemodynamic assessment, ensuring precise device placement, and detecting complications.

In the management of patients with stMCS devices, ultrasound plays a pivotal role beyond its established applications. Specifically, ultrasound guidance enhances the safety and success rate of vascular access procedures, which are critical in the placement and management of MCS devices. By providing real-time visualization of vascular anatomy, ultrasound reduces the risk of complications such as arterial puncture, haematoma, and catheter malposition, thereby improving patient outcomes.

Furthermore, ultrasound serves as a non-invasive tool to assess pulmonary congestion and systemic perfusion in patients supported by MCS devices. Lung ultrasound, for instance, can detect B-lines indicative of pulmonary oedema, allowing for timely interventions to manage fluid status and prevent respiratory complications. Additionally, Doppler ultrasound can evaluate systemic perfusion by assessing blood flow in various organs, aiding in the optimization of MCS device settings and overall patient management.^48^

However, several challenges and pitfalls impact its utility in critically ill patients. However, its implementation requires systematic imaging protocols, standardized interpretation and storage, and continuous education to maintain quality and clinical utility. The acquisition of Doppler-derived and myocardial performance indices is often challenging in patients with mechanical ventilation, post-cardiotomy status, anasarca, valvular disease, or atrial rhythm disorders, potentially limiting accurate assessment. Additionally, real-time quality assurance and imaging consistency remain concerns, particularly for handheld ultrasound devices, reinforcing the importance of interdisciplinary imaging expertise in critical care settings.

Given the complexity of stMCS management, integrating an imaging specialist into the multidisciplinary team is crucial to ensuring accurate assessments, guiding therapy adjustments, and optimizing clinical outcomes. Establishing clear diagnostic and monitoring protocols will improve clinical consistency, while validating advanced techniques, such as strain imaging and pressure-strain loops, could enhance patient selection for device de-escalation or transition to long-term support.

In conclusion, while echocardiography remains indispensable in stMCS management, ongoing research, validation, and standardization are essential to optimizing its role, refining patient selection, and improving clinical outcomes through AI, multimodality imaging, and echodynamic advancements.

## Supplementary Material

qyaf067_Supplementary_Data

## Data Availability

No new data were generated or analysed in support of this research.

## Lead author biography

Dr Hatem Soliman-Aboumarie is a consultant in Cardiothoracic Intensive Care, ECMO, and Mechanical Circulatory Support at Harefield Hospital, Royal Brompton and Harefield Hospitals, London. He leads Organ Donation and Advanced Critical Care Practitioners and is an honorary senior lecturer at King's College London. He serves as elected secretary and board member of the European Association of Cardiovascular Imaging (EACVI) and is on the EACVI Certification and Accreditation Committee. A fellow of the Royal College of Physicians (UK), FEACVI, FASE, and FHEA, he also sits on editorial boards and has edited key textbooks. He received the EACVI Excellence in Education Award in 2021.
