# Supplementary material for: Echocardiography for short-term mechanical circulatory support: a trans-Atlantic practical guide
Source: Eur Heart J Imaging Methods Pract. 2025 Jun 10;3(1):qyaf067. doi: 10.1093/ehjimp/qyaf067 (PMC12150027; doi:10.1093/ehjimp/qyaf067)
Supplement: qyaf067_Supplementary_Data [file qyaf067_supplementary_data.docx]

# **Supplementary material:**

**Table: stMCS configurations: summary of indications and some practical considerations**

| Device | Indication | Degree of support provided | Supporting part of the circulation | Insertion site | Oxygenation/ventilation support |
| --- | --- | --- | --- | --- | --- |
| IABP | Very limited; acute on chronic LV failure? May be used in mechanical complications | 0.5 – 1.0 L/min | LV (minimal afterload reduction) | Femoral artery – easy and quick access  Axillary artery – allows patient’s mobility | No |
| Impella CP | LV failure, active unloading | Max 3.8 L/min | LV | Femoral artery; easy and quick access | No |
| Impella 5.5 | LV failure, active unloading | Max 5.5 L/min | LV | Subclavian artery (surgical insertion) | No |
| Impella RP/RP Flex | RV failure, RV unloading | Max 3.8 L/min | RV | Femoral vein and inferior vena cava; easy and quick access  Internal jugular vein and superior vena cava | No |
| Protek Duo | RV failure | Max 4-5 L/min | RV | Jugular vein | Yes |
| Peripheral V-A ECMO | Biventricular failure | Max 7 L/min | RV and LV | Fem/jug. Vein to fem/subcl artery | Yes |
| BiPella | RV and LRV failure, active unloading | 3.8-4.2 L/min | RV and LV | Femoral artery and vein; easy and quick access | No |
| ECMELLA (or ECPELLA): V-A ECMO plus Impella CP or 5.5 | Biventricular failure, need for active LV unloading | V-A ECMO: max 7 L/min, Impella CP: 3.8 L/min, Impella 5.5: 5.5 L/min | RV and LV | Femoral vein (or jugular vein), femoral artery (or axillary artery) for V-A; CP: femoral artery, 5.5: Subclavian artery (surgical insertion) | Yes |

**Overview of Short-term Mechanical Circulatory Support (stMCS) devices**

A variety of short-term (temporary) mechanical circulatory support devices are available to manage cardiogenic shock, each with distinct mechanisms, indications, and configurations. Below is a brief summary of commonly used stMCS devices and their key characteristics:

**a)** **Intra-aortic balloon pump (IABP)**

The IABP supports patients with cardiogenic shock based on the principle of counterpulsation. Counterpulsation refers to balloon inflation during diastole and deflation in early systole, which displaces blood in the aorta, potentially increasing coronary blood flow and systemic perfusion.

1. **Micro-axial flow devices**

Micro-axial flow pumps (mAFPs) are small, percutaneously inserted cardiac supported devices placed across the aortic or tricuspid valve. They directly unload the ventricle and provide continuous flow in the systemic or pulmonary circulation. The most typical mAFP pumps are the Impella devices (Abiomed), with both left and right support systems currently available.

1. **The ProtekDuo dual-lumen cannula**

The ProtekDuo dual-lumen cannula is a temporary percutaneous right ventricular assist device (RVAD) which provides a veno-pulmonary ECMO (V-P ECMO) support, offering a complete bypass of the RV and is particularly suited for patients with primary isolated RV dysfunction with or without respiratory failure

1. **V-A ECMO**

V-A ECMO is a simplified cardiopulmonary bypass circuit and consists of an oxygenator, pump, a heater/cooler and large bore tubing and cannulas. Circuit configuration can vary but a uniform outline for V-A ECMO is: venous blood is drained from the inferior vena cava (IVC) and right atrium outside the body, is then oxygenated, warmed and pumped back into the arterial system either through the ascending aorta (central V-A ECMO) or through the femoral artery (peripheral V-A ECMO). V-A ECMO bypasses the cardiopulmonary circulation, so pulmonary blood flow and left ventricular (LV) preload are decreased while LV afterload is increased because of the pressurised return of blood via the arterial cannula.

1. **Combined configurations**

The BiPella strategy combines the Impella RP with the Impella CP/5.5. This technique can be used in cases of biventricular failure (or in initial LV failure with the need for additional RV support) in patients who do not require supplementary pulmonary support. The ECMELLA strategy combines peripheral V-A ECMO with the percutaneous Impella CP strategy. The pathophysiological basis of ECMELLA is centred around mitigating peripheral V-A ECMO-related side effects (ECMO-induced high LV afterload) and the additional benefit of myocardial unloading.
